# Development and Validation of an LC–MS/MS Method for Simultaneous Quantification of Pazopanib, Cabozantinib, Sorafenib, Axitinib, Tivozanib, Sunitinib, and the Metabolites Sorafenib N-Oxide and Desethyl-Sunitinib in Human Plasma

**DOI:** 10.3390/ph19081180

**Published:** 2026-07-28

**Authors:** Eva Greibe, Jakob N. Henriksen, Niels Fristrup, Elke Hoffmann-Lücke

**Affiliations:** 1 Department of Clinical Biochemistry, Aarhus University Hospital, 8200 Aarhus N, Denmark; elkehoff@rm.dk; 2 Department of Clinical Medicine, Aarhus University, 8000 Aarhus, Denmark; 3 Department of Clinical Pharmacology, Aarhus University Hospital, 8200 Aarhus N, Denmark; jakob.n.henriksen@biomed.au.dk (J.N.H.); niels.fristrup@rm.dk (N.F.); 4 Department of Oncology, Aarhus University Hospital, 8200 Aarhus N, Denmark

**Keywords:** pazopanib, cabozantinib, sorafenib, axitinib, tivozanib, sunitinib, sorafenib N-oxide, desethyl-sunitinib, tyrosine kinase inhibitor, stability, renal cell carcinoma

## Abstract

**Background:** Pazopanib, cabozantinib, sorafenib, axitinib, tivozanib, and sunitinib are widely used tyrosine kinase inhibitors (TKIs) targeting the vascular endothelial growth factor receptor signaling pathway. Considerable interindividual pharmacokinetic variability, together with established exposure–toxicity relationships, underlines the need for reliable analytical methods for therapeutic drug monitoring (TDM). The aim of this study was to develop and validate an LC–MS/MS method for the simultaneous determination of these six TKIs and the two metabolites, sorafenib N-oxide and desethyl-sunitinib, in human plasma and to investigate their stability under relevant preanalytical conditions. **Methods:** Plasma samples were prepared by protein precipitation and analyzed using LC–MS/MS with positive electrospray ionization and multiple reaction monitoring. Validation included assessments of linearity, accuracy, precision, lower limit of quantification, dilution integrity, carry-over, and stability under different storage conditions. As proof of concept, the method was applied to plasma samples from patients receiving TKI treatment. **Results:** All analytes demonstrated linear responses across the validated calibration ranges. Validation parameters met predefined acceptance criteria for all analytes, except for sorafenib N-oxide, which exhibited CV% values slightly above the acceptance limit (15%). All compounds remained stable during storage at −80 °C for at least five years. Furthermore, all analytes were stable at room temperature for 24 h, except sorafenib N-oxide, which showed instability under these conditions. Analysis of patient samples confirmed the suitability of the method for clinical application. **Conclusions:** We established and validated a fast, sensitive, and accurate LC–MS/MS method enabling simultaneous quantification of six TKIs and two metabolites in human plasma. The analytical method was successfully applied to plasma samples from patients in treatment with the six TKIs, demonstrating proof of concept for its application in TDM.

## 1. Introduction

Tyrosine kinase inhibitors (TKIs) targeting the vascular endothelial growth factor receptor (VEGFR) pathway—including pazopanib, cabozantinib, sorafenib, axitinib, tivozanib, and sunitinib—have transformed the management of several malignancies, including metastatic renal cell carcinoma [1,2]. Despite their clinical benefits, treatment responses vary widely among patients due to considerable interindividual variability in pharmacokinetics, metabolism, and drug exposure [3].

Multiple studies have demonstrated exposure–toxicity relationships for TKIs [4,5,6,7] and, for several drugs, also exposure–efficacy correlations [8,9,10]. These findings highlight the potential clinical benefit of therapeutic drug monitoring (TDM) to individualize dosing and improve treatment outcomes.

Many VEGFR-TKIs form clinically relevant active metabolites, including sorafenib (sorafenib N-oxide metabolite) and sunitinib (desethyl-sunitinib metabolite), which contribute to therapeutic and adverse effects. Several LC–MS/MS methods have been reported for individual VEGFR-targeting TKIs, selected metabolites, or broader panels of kinase inhibitors [11,12,13,14,15,16]. For example, Li et al. [11] and Rais et al. [13] described assays including sorafenib N-oxide and desethyl-sunitinib, respectively, whereas multiplex methods by Herbrink et al. [12], Bouchet et al. [15], and Chen et al. [16] enabled simultaneous quantification of multiple kinase inhibitors. However, to our knowledge, no published method has combined the six VEGFR-targeting TKIs included in the present study with two clinically relevant metabolites, sorafenib N-oxide and desethyl-sunitinib, within a single analytical workflow. Furthermore, limited information is available regarding the long-term stability of these analytes under storage conditions relevant for therapeutic drug monitoring and biobanking. Therefore, the aim of this study was to develop and validate a robust LC–MS/MS method for simultaneous quantification of pazopanib, cabozantinib, sorafenib, axitinib, tivozanib, and sunitinib and the active metabolites sorafenib N-oxide and desethyl-sunitinib in human plasma. An additional aim was to evaluate analyte stability under storage and handling conditions relevant for clinical studies, therapeutic drug monitoring, and biobanking, including long-term storage at −80 °C for up to five years.

## 2. Results and Discussion

This study presents a novel and validated LC-MS/MS method for the simultaneous quantification of pazopanib, cabozantinib, sorafenib and its metabolite sorafenib N-oxide, axitinib, tivozanib, sunitinib, and its metabolite desethyl-sunitinib in human plasma. In addition, new data on stability are reported.

The chromatography method was developed and optimized using a trial-and-error approach. Both positive and negative electrospray ionization modes were evaluated for the detection of each analyte. All compounds demonstrated adequate ionization efficiency in positive ionization mode and were therefore analyzed under these conditions. Precursor and product ions were selected for each analyte, and maximal ion abundance was achieved through optimization of source, gas, and compound-dependent parameters. The optimal ionization conditions are summarized in Table 1. Internal standards (ISs) were used for relative quantification, reducing errors caused by differences in injection volume, ionization, and chromatographic retention time variance.

**Figure 1:**
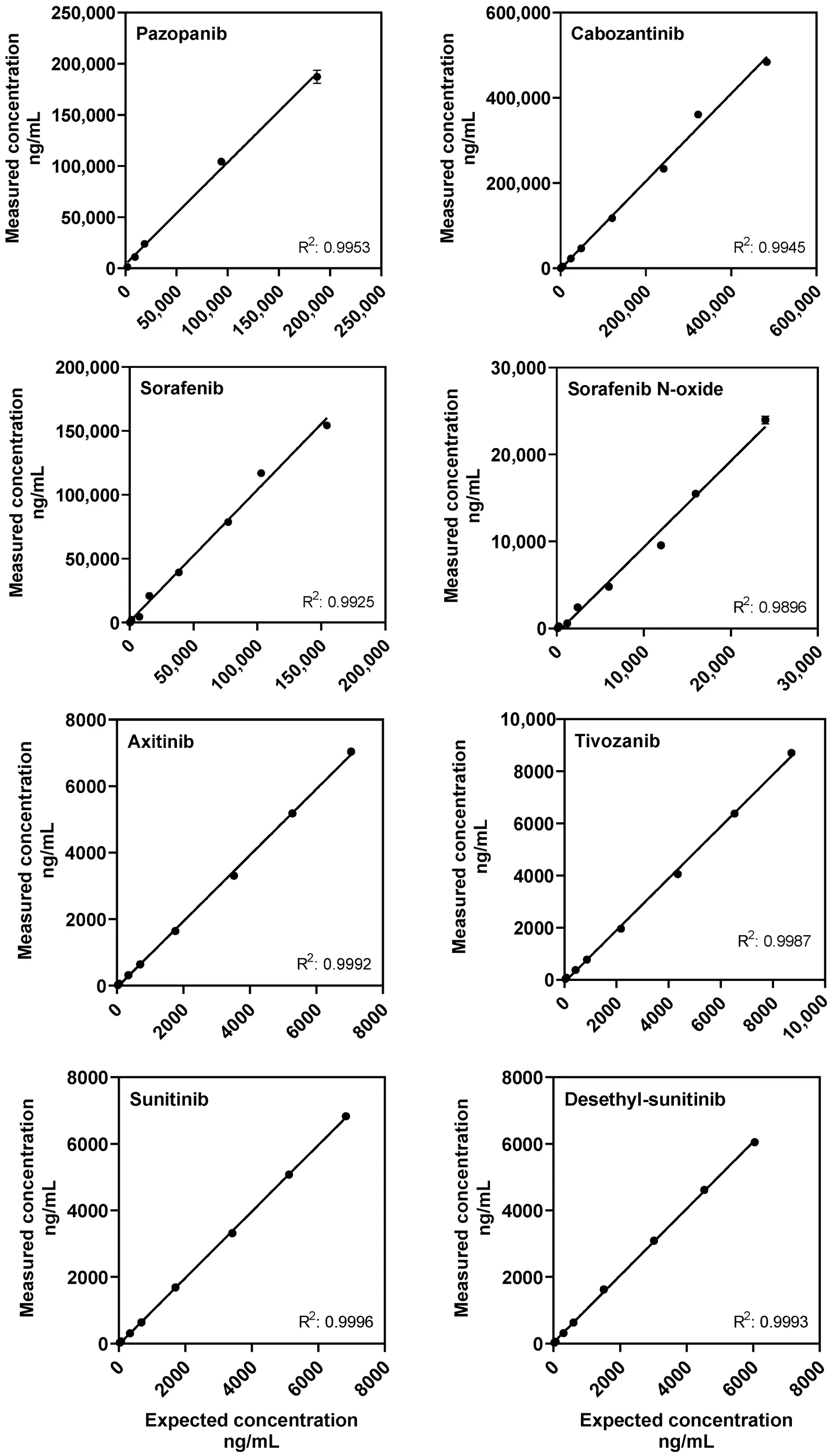
Linearity based on dilution series. Linearity was tested by analyzing dilution series in triplicate. The plotted replicates (*n* = 3) demonstrated satisfactory dilution integrity and linearity (R^2^ > 0.98) from LLOQ to at least 180,000 ng/mL (pazopanib), 480,000 ng/mL (cabozantinib), 150,000 ng/mL (sorafenib), 23,000 ng/mL (sorafenib N-oxide), 7000 ng/mL (axitinib), 8000 ng/mL (tivozanib), 7000 ng/mL (sunitinib), and 6000 ng/mL (desethyl-sunitinib).

In each validation batch, six calibration standards were analyzed, and the resulting calibration curves demonstrated linear responses across the specified concentration range (Table 2). The squared correlation coefficients (R^2^) for all calibration curves were ≥0.98, indicating good linearity within the calibration range. Linearity beyond the upper limit of the calibration range was assessed by performing a dilution series. The diluted replicates demonstrated acceptable dilution integrity and maintained linearity (R^2^ > 0.98), as illustrated in Figure 1.

For carry-over, the peak areas of the blank plasma samples at the retention time of the analytes had to be below the lower limit of quantification (LLOQ) peak area. No carry-over was found at 100,000 ng/mL (pazopanib), 5000 ng/mL (cabozantinib), 4500 ng/mL (sorafenib), 2400 ng/mL (sorafenib N-oxide), 5000 ng/mL (axitinib), 2000 ng/mL (tivozanib), 3300 ng/mL (sunitinib), and 4500 ng/mL (desethyl-sunitinib).

The inter-assay precision and accuracy of the TKIs and their metabolites were evaluated at four concentration levels (LLOQ, Low QC, Middle QC, and High QC). Overall, the analytes demonstrated low inter-assay variability and fulfilled the predefined validation acceptance criteria for all analytes except sorafenib N-oxide (Table 3). For sorafenib N-oxide, the precision criterion was exceeded at the LLOQ, and CV values were slightly above the predefined acceptance limit at the low QC level. Although this indicates reduced assay performance at the lower end of the calibration range, the method is considered fit for its intended clinical application, as reported plasma concentrations of sorafenib N-oxide during sorafenib treatment [2,6] are typically well above the validated LLOQ and within the concentration range where acceptable precision and accuracy were achieved. Nevertheless, concentrations close to the LLOQ should be interpreted with caution. If low concentration levels become clinically relevant for TDM, lower-dose regimens, or other clinical indications, the lower end of the measuring range should be optimized to ensure reliable quantification in the relevant concentration range.

The stability of the analytes in human plasma under various storage temperatures and time conditions and in the autosampler was evaluated using two independent stock solutions for each analyte. The results are summarized in Table 4. Pazopanib, cabozantinib, sorafenib, axitinib, tivozanib, sunitinib, and desethyl-sunitinib were stable in plasma for 24 h both under benchtop conditions at room temperature and when stored in the autosampler at 10 °C after post-preanalytical handling. Furthermore, all analytes remained stable during storage at −80 °C for five weeks and after three freeze–thaw cycles. In contrast, sorafenib N-oxide was unstable at room temperature after 24 h (RE% ≥ 15%), which was consistent with previous reports describing instability under ambient conditions [11]. Overall, the observed stability patterns were in agreement with findings reported in the literature [11,12,13,15]. To our knowledge, this study is the first to evaluate the long-term stability of these analytes in plasma stored at −80 °C for up to five years. Notably, all analytes remained stable throughout the investigated long-term storage period.

Compared with previously published LC–MS/MS methods for therapeutic drug monitoring of TKIs, the present assay offers several distinguishing features. Multiplex assays have enabled simultaneous quantification of multiple TKIs [12,15,16], whereas individual assays have reported quantification of sorafenib together with sorafenib N-oxide [11] or sunitinib together with desethyl-sunitinib [13]. To our knowledge, no published method has combined the six VEGFR-targeting TKIs included in the present study with both clinically relevant metabolites, sorafenib N-oxide and desethyl-sunitinib, within a single analytical workflow. This enables assessment of both parent drug exposure and metabolite formation across a focused panel of VEGFR-targeting agents used in renal cell carcinoma.

The analytical workflow is based on straightforward protein precipitation without solid-phase extraction or evaporation steps, facilitating implementation in routine LC-MS/MS laboratories. In comparison, Bouchet et al. employed solid-phase extraction prior to LC-MS/MS analysis [15]. The total run time of six minutes further supports high-throughput sample analysis while maintaining robust analytical performance. Furthermore, although stability data have previously been reported for individual analytes and shorter storage periods [11,13,15], we are not aware of previous studies evaluating the long-term stability of all included analytes after five years of storage at −80 °C. This finding is particularly relevant for retrospective pharmacokinetic studies and biobanking initiatives where archived plasma samples may be analyzed years after collection.

**Figure 2:**
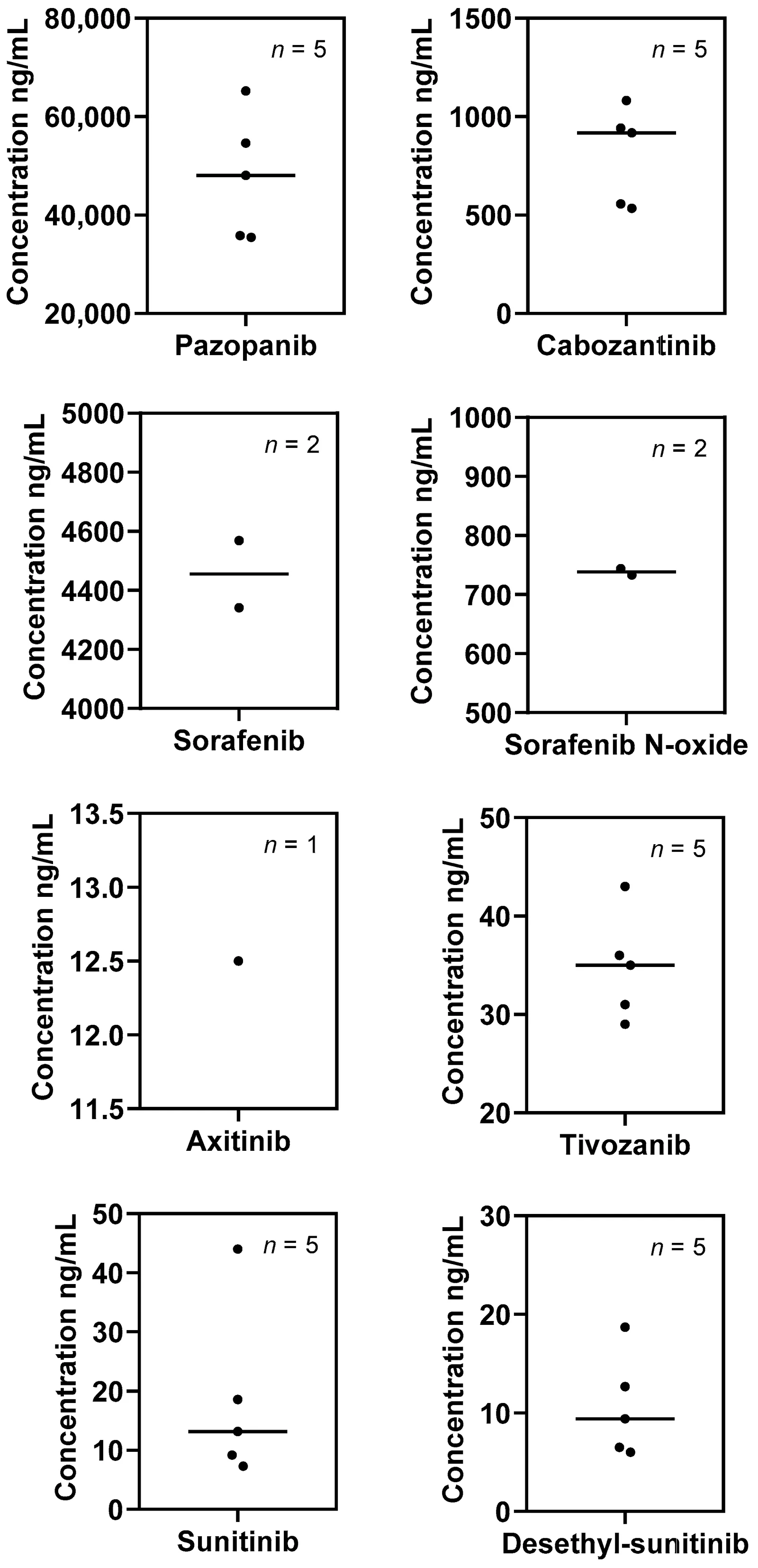
Method application in a clinical setting. Plasma concentration of pazopanib (200–600 mg/day), cabozantinib (20–100 mg/day), sorafenib (200–400 mg twice a day), sorafenib N-oxide, axitinib (5 mg twice a day), tivozanib (890–1340 µg/day), sunitinib (37.5–50 mg/day), and desethyl-sunitinib from metastatic renal cell carcinoma patients in treatment with the respective TKIs. Results are shown as individual values with medians. The figure was made in GraphPad Prism version 8.

To demonstrate the clinical application of the method, plasma samples from patients with metastatic renal cell carcinoma receiving treatment with the included TKIs were analyzed for concentrations of the parent drugs and the metabolites. The measured plasma concentrations (Figure 2) were within the ranges previously reported in the literature [2]. No formal assessment of therapeutic target attainment was performed.

Beyond its analytical characteristics, the method offers several practical advantages for clinical implementation. The combination of simple sample preparation and a short chromatographic run time enables reporting of results within 24 h after sample receipt, making the assay compatible with routine TDM. Such turnaround times may facilitate timely dose adjustments in patients receiving TKIs. Rapid availability of plasma concentration data is particularly relevant in oncology, where treatment efficacy and toxicity are closely linked to drug exposure, and early pharmacokinetic information may support individualized dosing strategies.

The median concentration of sorafenib N-oxide (739 [733–744] ng/mL, *n* = 5) was approximately six-fold lower than that of sorafenib (4455 [4341–4569] ng/mL), corresponding to a metabolite-to-parent drug ratio of approximately 16%. This finding is consistent with previous reports by Li et al. [11], who demonstrated that sorafenib represents approximately 70–85% of circulating analytes, whereas sorafenib N-oxide accounts for approximately 9–17% at steady state. The observed ratio in the present study therefore falls within the upper end of the range reported in the literature, supporting the applicability of the method to clinical samples. The relatively lower abundance of sorafenib N-oxide compared with the parent compound is expected, as sorafenib N-oxide is formed through CYP3A4-mediated metabolism and constitutes the major, but quantitatively smaller, circulating metabolite of sorafenib. Despite the limited sample size, the concentration pattern observed in the present study is in agreement with previously reported pharmacokinetic data, indicating that the method provides reliable quantification of both sorafenib and sorafenib N-oxide in clinical plasma samples. The median concentrations of sunitinib (13.2 [7.3–44] ng/mL, *n* = 5) and its metabolite desethyl-sunitinib (9.4 [6.0–12.7] ng/mL) were found to be relatively similar, indicating comparable exposure to the parent drug and metabolite. This observation differs from the findings reported by Numakura et al. [17], who observed substantially higher plasma concentrations of sunitinib (76.7 ± 39.8 ng/mL) compared with desethyl-sunitinib (16.8 ± 9.5 ng/mL), corresponding to an approximately 4.5-fold higher concentration of the parent compound. In contrast, the ratio between sunitinib and desethyl-sunitinib in the present study was approximately 1.5. As both studies evaluated trough plasma concentrations, the observed differences are unlikely to be explained by sampling time alone and may instead reflect differences in patient characteristics, dosing regimens, treatment duration, or interindividual metabolic variability. Several factors may explain this discrepancy, including differences in patient populations, dosing regimens, sampling strategies, treatment duration, and interindividual variability in CYP3A4-mediated metabolism. Furthermore, the small sample size of the present study limits direct comparison with larger clinical cohorts. Nevertheless, both studies demonstrated quantifiable concentrations of sunitinib and desethyl-sunitinib in patient plasma, supporting the applicability of the present LC–MS/MS method for pharmacokinetic studies and TDM of TKIs.

This study has several limitations that should be considered when interpreting the findings. First, isotopically labeled ISs were not available for cabozantinib, tivozanib, sorafenib N-oxide, and desethyl-sunitinib at the time of method development. Consequently, these analytes may be more susceptible to matrix-related ion suppression or enhancement than analytes corrected with structurally identical ISs. In addition, the ISs were added after protein precipitation, meaning that variability introduced during sample extraction could not be fully compensated. Matrix effect and recovery experiments were not performed and therefore represent a limitation of the present study. Nevertheless, the generally acceptable validation performance for all analytes, with the exception of sorafenib N-oxide at the lower end of the calibration range, suggests that these methodological limitations had only a limited impact under the analytical conditions employed. The assay is therefore considered suitable for its intended application in TDM, although future studies should include dedicated matrix effect and recovery experiments and incorporate isotope-labeled internal standards for all analytes where available. Second, the patient cohort included a limited number of samples, particularly for sorafenib, axitinib, and the metabolite analyses, which limits the generalizability of the clinical observations. Consequently, the clinical application presented in this study should be regarded as a proof of concept demonstrating the real-world feasibility of the validated assay rather than evidence of the clinical utility of TDM. Larger prospective studies including more diverse patient populations are warranted to evaluate the clinical impact of implementing the assay in routine TDM. Finally, quality control samples and calibration standards were prepared from the same stock solutions. Although this approach ensured consistency during method development and validation, it may have underestimated total analytical variability and could potentially overestimate method accuracy and precision. Nevertheless, the generally acceptable validation performance suggests that this limitation had only a limited impact under the analytical conditions employed.

## 3. Materials and Methods

An LC-MS/MS multiple reaction monitoring (MRM) method for quantification of pazopanib, cabozantinib, sorafenib, axitinib, tivozanib, sunitinib, and the metabolites sorafenib N-oxide and desethyl-sunitinib in human plasma was developed. The methodological details are outlined below.

### 3.1. Chemicals and Reagents

A stock solution (IVD, CE marked) of pazopanib (MW 437.52 g/mol), cabozantinib (MW 501.51 g/mol), sorafenib (MW 464.82 g/mol), sorafenib N-oxide (MW 480.82 g/mol), axitinib (MW 386.47 g/mol), tivozanib (MW 454.86 g/mol), sunitinib (MW 398.48 g/mol), and desethyl-sunitinib (MW 370.42 g/mol) in human serum were obtained from UTAK Laboratories (Valencia, CA, USA).

Isotopically labeled ISs (pazopanib-d3, sorafenib-d3, axitinib-d3, and sunitinib-d4) were purchased from Toronto Research Chemicals (Vaughan, ON, Canada). It was not possible to purchase ISs for cabozantinib, tivozanib, and the two metabolites at the time.

LC–MS grade water, methanol, and acetonitrile were purchased from VWR International (Soeborg, Denmark). Ammonium acetate was purchased from Sigma-Aldrich (Soeborg, Denmark), and formic acid was purchased from Merck (Soeborg, Denmark).

### 3.2. Preparation of Calibration Standards and QC Samples

The purchased serum stock solution from UTAK Laboratories contained a mixture of all eight compounds. This stock was stored at −80 °C and used for the preparation of standard calibration samples and quality controls in plasma.

The final concentrations of the standard calibration samples were as follows: pazopanib (0 (blank), 2500, 5000, 12,500, 25,000, 37,500, and 50,000 ng/mL); cabozantinib and sorafenib (0 (blank), 500, 1000, 2500, 5000, 7500, and 10,000 ng/mL); and sorafenib N-oxide, axitinib, tivozanib, sunitinib, and desethyl-sunitinib (0 (blank), 50, 100, 250, 500, 750, and 1000 ng/mL).

The final concentrations of the quality controls were as follows: pazopanib: Low QC: 10,000 ng/mL, Middle QC: 16,000 ng/mL, and High QC: 33,000 ng/mL; cabozantinib and sorafenib: Low QC: 2000 ng/mL, Middle QC: 3300 ng/mL, and High QC: 7500 ng/mL; sorafenib N-oxide: Low QC: 200 ng/mL, Middle QC: 350 ng/mL, and High QC: 700 ng/mL; and axitinib, tivozanib, sunitinib, and desethyl-sunitinib: Low QC: 200 ng/mL, Middle QC: 350 ng/mL, and High QC: 500 ng/mL.

The IS stock solutions (pazopanib-d3, sorafenib-d3, axitinib-d3, and sunitinib-d4) were prepared in methanol at a concentration of 1 mg/mL and stored at −80 °C. An IS working solution was prepared by combining appropriate volumes of the IS stock solutions and diluting the resulting mixture 1:1000 (*v*/*v*) with methanol–water. The IS working solution was stored at 4 °C until use.

### 3.3. Sample Pretreatment

Due to differences in expected plasma concentration ranges, samples intended for the analysis of pazopanib, cabozantinib, sorafenib, and sorafenib N-oxide were diluted 1:800, whereas samples for axitinib, tivozanib, sunitinib, and desethyl-sunitinib were diluted 1:160. The sample pretreatment procedures were as follows:

Plasma samples (50 µL EDTA plasma) were mixed with 150 µL acetonitrile, vortexed, incubated for 5 min, and centrifuged at 10,000× *g* for 10 min. A volume of 150 µL of the resulting supernatant was transferred to a clean tube and diluted 1:2 by adding 150 µL of a methanol–water mixture (1:1, *v*/*v*). For the analysis of pazopanib, cabozantinib, sorafenib, and sorafenib N-oxide, 10 µL of this dilution was further diluted 1:50 with 490 µL methanol–water (1:1, *v*/*v*). For the analysis of axitinib, tivozanib, sunitinib, and desethyl-sunitinib, 100 µL of the initial 1:2 dilution was instead diluted 1:10 with 900 µL methanol–water (1:1, *v*/*v*). Subsequently, 100 µL of the resulting solution was mixed 1:2 with 100 µL of the IS working solution in light-protected microtubes and incubated for 5 min at 70 °C in a water bath. The overall dilution factors resulting from the sample preparation procedure were 1:800 for pazopanib, cabozantinib, sorafenib, and sorafenib N-oxide and 1:160 for axitinib, tivozanib, sunitinib, and desethyl-sunitinib. A volume of 3 µL was injected into the HPLC system.

### 3.4. Instrumentation

Liquid chromatography was performed on an Agilent 1290 Infinity Series System (Agilent, Glostrup, Denmark), and mass spectrometric detection was carried out on an Agilent 6470A Triple Quad mass spectrometer (Agilent), equipped with an electrospray ionization (ESI) source operated in positive mode. Chromatographic separation was performed on a Kinetex^®^ EVO C18 column (2.1 × 150 mm, 2.6 µm) from Phenomenex (Copenhagen, Denmark) maintained at 35 °C.

### 3.5. Liquid Chromatography and Mass Spectrometry

Mobile phases consisted of A: 10 mM ammonium acetate with 0.1% formic acid and B: acetonitrile. The gradient was 0 min, 10% B; 3.5 min, 90% B; and 5 min, 10% B. Flow rate was 0.4 mL/min, and the total run time was 6 min. MRM transitions were optimized individually for each analyte and IS (Table 1). Cell accelerator voltage was 4 V for all compounds. Gas temperature was 260 °C with a flow of 13 L/min; nebulizer was 60 psi; and sheath gas temperature was 400 °C with a flow of 12 L/min. Capillary voltage was 3500 V, and nozzle voltage was 0 V.

### 3.6. Data Analysis

Peak area ratios (analyte/IS) were used for linear regression. Calibration curves were accepted if all non-zero points were within ±15% of nominal values and R^2^ ≥ 0.98.

### 3.7. Method Validation

For validation of the developed method, linearity, dilution integrity, carry-over, LLOQ, precision (CV%), accuracy, and stability were assessed.

Calibration standards and quality control samples were prepared from the same stock solution. Although this ensured consistency during method development and validation, the use of a common stock solution may underestimate total analytical variability and could potentially overestimate method accuracy and precision compared with independently prepared calibration standards and quality control samples. This limitation should be considered when interpreting the reported accuracy and precision.

#### 3.7.1. Calibration Curves, Linearity, and Dilution Integrity

The linearity of the developed methods was evaluated across their respective calibration ranges, as outlined in Section 3.2. Calibration curves were constructed by plotting the peak area ratios of the analyte against the IS. Each curve consisted of six non-zero calibration standards and a blank sample. Acceptance criteria for the calibration curves are described in Section 3.6.

Linearity above the upper limit of the calibration range was assessed using high-concentration stock solutions of the respective TKIs, which were used to prepare a dilution series (100%, 75%, 50%, 10%, 5%, 1%, 0.5%, and 0.1%). Each level was analyzed in triplicate and evaluated by linear regression analysis. A squared correlation coefficient (R^2^) greater than 0.98 was considered indicative of acceptable linearity.

#### 3.7.2. Carry-Over

Carry-over was evaluated in conjunction with the linearity experiments by injecting three blank plasma samples after each concentration level in the dilution series. To exclude carry-over, the analyte response in the blank samples at the corresponding retention time was required to be below the LLOQ. The LLOQ was established as described in Section 3.7.3.

#### 3.7.3. Precision and Accuracy

During validation, method precision and accuracy were evaluated at two concentration levels (Low QC and Middle QC) by analyzing each control 12 times as a single determination. The validation criteria were that the inter-assay precision (CV%) and the deviation from the nominal concentration (relative error (RE%)) should be ≤15%. Precision and accuracy were also determined based on QC measurements from 19 runs obtained during the analysis of project samples [18]. For these analyses, a third QC level (High QC) was included in the method. The LLOQ was established by analyzing five replicates across four independent runs (*n* = 20) at a concentration level well below the clinically relevant concentration range for each TKI or metabolite. The validation criteria for the LLOQ required signal-to-noise ratio (S/N) ≥ 10, CV% ≤ 20%, and RE% ≤ 15%.

#### 3.7.4. Stability

Stability was assessed under different storage and handling conditions using two independently prepared plasma stocks for each analyte, representing the low and middle QC concentration levels. Baseline concentrations were determined immediately after preparation of each stock. Aliquots from each stock were then assigned to the different stability conditions, including three freeze–thaw cycles, bench-top storage, autosampler storage, deep-freeze storage, and long-term storage. Stability was evaluated by comparing the measured concentration after each storage condition with the corresponding baseline concentration obtained from the same stock, and results are expressed as relative error (RE%). Freeze–thaw stability was evaluated after three freeze (−80 °C) and thaw (room temperature) cycles. Bench-top stability was assessed after 24 h of storage at room temperature. Autosampler stability was assessed after 24 h in the autosampler at 10 °C (post-preanalytical handling). Deep-freeze stability was investigated after storage at −80 °C for five weeks. Long-term stability was assessed after five years at −80 °C. For all stability tests, the acceptance criterion was a deviation from baseline concentration (RE%) of ≤15%.

### 3.8. Clinical Application

The validated method was applied to plasma samples to demonstrate usability for a clinical setting and proof of concept. Plasma from metastatic renal carcinoma patients treated with pazopanib (200–600 mg/day, *n* = 5), cabozantinib (20–100 mg/day, *n* = 5), sorafenib (200–400 mg twice a day, *n* = 2), axitinib (5 mg twice a day, *n* = 1), tivozanib (890–1340 µg/day, *n* = 5), and sunitinib (37.5–50 mg/day, *n* = 5) at Aarhus University Hospital, Denmark, were used to test the methods in a clinical setting. The inclusion of patients was part of a larger project [18] and followed ethical guidelines, including the Helsinki Declaration and Danish law. The samples were collected in EDTA blood-collecting tubes, and plasma was stored at −80 °C until analysis.

## 4. Conclusions

A robust LC–MS/MS method was developed and validated for the simultaneous determination of pazopanib, cabozantinib, sorafenib, axitinib, tivozanib, sunitinib, and the metabolites sorafenib N-oxide and desethyl-sunitinib in human plasma. To our knowledge, this study is among the first to report a fully validated LC–MS/MS assay enabling concurrent quantification of these eight analytes and the first to provide long-term stability data for plasma samples stored at −80 °C for five years. These results strengthen confidence in long-term sample storage for retrospective pharmacokinetic investigations and clinical studies, thereby supporting reliable analyses in multicenter trials and biobanking. Furthermore, our observations, together with previous reports describing the instability of sorafenib N-oxide at room temperature, indicate that plasma samples should preferably be handled and processed on ice. Overall, the validated method represents a rapid, sensitive, and reliable analytical approach suitable for pharmacokinetic applications and with potential utility in clinical TDM.

## Figures and Tables

**Table 1:** Optimized mass spectrometric parameters for analytes and ISs.

Compound	Precursor Ion, *m*/*z*	Product Ion, *m*/*z*	Fragmentor Voltage, V	Collision Energy, V
Cabozantinib	502.0	391.0	220	40
Pazopanib	438.0	357.0	200	35
Pazopanib-d3 (IS)	441.0	360.0	200	35
Sorafenib	465.1	252.0	180	42
Sorafenib-d3 (IS)	468.1	211.1	180	42
Sorafenib N-oxide	481.0	286.0	180	30
Axitinib	387.0	356.0	120	20
Axitinib-d3 (IS)	390.0	356.1	120	20
Tivozanib	455.0	341.0	140	58
Sunitinib	399.0	326.0	130	23
Sunitinib-d4 (IS)	403.0	330.0	130	23
Desethyl-sunitinib	371.0	283.0	100	30

Abbreviations: internal standard (IS).

**Table 2:** Linearity as judged from the equation of calibration curves ^a^.

Compound	Calibration Range (ng/mL) (from LLOQ)	Regression Equation (y = ax + b)	Correlation Coefficient (R^2^)
Pazopanib	300–50,000	y = 0.00005x + 0.00689	0.9996
Cabozantinib	100–10,000	y = 0.00001x + 0.00002	0.9997
Sorafenib	75–10,000	y = 0.00094x + 0.08172	0.9997
Sorafenib N-oxide	100–1000	y = 0.00037x + 0.00361	0.9992
Axitinib	5–1000	y = 0.00045x + 0.00132	0.9999
Tivozanib	5–1000	y = 0.00028x + 0.00008	0.9997
Sunitinib	5–1000	y = 0.00240x − 0.00732	0.9998
Desethyl-sunitinib	5–1000	y = 0.00333x − 0.00270	0.9996

^a^ All calibration standards were within ±15% of their nominal concentration, and the squared correlation coefficients (R^2^) of the calibration curves were ≥0.98 in each analytical run.

**Table 3:** Total inter-assay precision and accuracy in plasma.

Analyte	LLOQ (*n* = 20)	Low QC (*n* = 30)	Middle QC (*n* = 30)	High QC (*n* = 19)
**Pazopanib—conc. ^a^**	**300 ng/mL**	**10,000 ng/mL**	**16,000 ng/mL**	**33,000 ng/mL**
Mean (ng/mL)	297.2	10,385.2	17,198.2	35,192.5
CV% ^b^	5.8	3.9	2.9	14.5
RE% ^c^	−0.9	3.9	7.5	6.6
**Cabozantinib—conc.**	**100 ng/mL**	**2000 ng/mL**	**3300 ng/mL**	**7500 ng/mL**
Mean (ng/mL)	112.6	2118.8	3474.0	7560.7
CV%	14.2	4.9	4.9	10.1
RE%	12.6	5.9	5.3	0.8
**Sorafenib—conc.**	**75 ng/mL**	**2000 ng/mL**	**3300 ng/mL**	**7500 ng/mL**
Mean (ng/mL)	71.8	2135.5	3457.4	7341.4
CV%	14.1	4.7	2.8	7.6
RE%	−4.2	6.8	4.8	−2.1
**Sorafenib N-oxide—conc.**	**100 ng/mL**	**200 ng/mL**	**350 ng/mL**	**700 ng/mL**
Mean (ng/mL)	97.8	213.9	348.8	707.8
CV%	30.5	16.2	16.4	14.9
RE%	−2.2	6.9	−0.3	1.1
**Axitinib—conc.**	**5 ng/mL**	**200 ng/mL**	**350 ng/mL**	**500 ng/mL**
Mean (ng/mL)	5.7	207.5	349.0	537.2
CV%	10.2	3.2	3.4	10.9
RE%	13.5	3.7	−0.3	7.4
**Tivozanib—conc.**	**5 ng/mL**	**200 ng/mL**	**350 ng/mL**	**500 ng/mL**
Mean (ng/mL)	5.8	209.0	348.1	538.6
CV%	13.0	5.2	4.8	13.6
RE%	15.4	4.5	−0.5	7.7
**Sunitinib—conc.**	**5 ng/mL**	**200 ng/mL**	**350 ng/mL**	**500 ng/mL**
Mean (ng/mL)	5.3	202.8	339.5	524.7
CV%	10.2	3.9	3.0	9.8
RE%	5.2	1.4	−3.0	4.9
**Desethyl-sunitinib—conc.**	**5 ng/mL**	**200 ng/mL**	**350 ng/mL**	**500 ng/mL**
Mean (ng/mL)	5.4	205.2	344.0	541.8
CV%	7.8	4.5	2.7	9.5
RE%	7.5	2.6	−1.7	8.4

^a^ Nominal concentrations are given in bold font; ^b^ CV% [SD/mean] × 100 (accept criterion QC level: CV% ≤ 15%; accept criterion LLOQ level: CV% ≤ 20%); ^c^ RE% (relative error expressed as [mean observed concentrations—nominal concentration)/(nominal concentration)] × 100 (accept criterion RE% ≤ 15%).

**Table 4:** Stability assessment of analytes in plasma.

Analyte	Stability Test	Stock 1 Nominal Conc., ng/mL	Stock 1 RE% ^f^	Stock 2 Nominal Conc., ng/mL	Stock 2 RE% ^f^
Pazopanib	Freeze–thaw ^a^	10,000	−10.1	16,000	−5.9
	Autosampler ^b^		−6.3		−6.5
	Bench-top ^c^		−8.4		−2.7
	Deep-freeze ^d^		−7.3		−0.8
	Long-term ^e^		8.4		−7.2
Cabozantinib	Freeze–thaw ^a^	2000	9.7	3300	12.0
	Autosampler ^b^		5.5		−1.8
	Bench-top ^c^		−2.5		12.9
	Deep-freeze ^d^		7.4		3.1
	Long-term ^e^		1.1		−3.6
Sorafenib	Freeze–thaw ^a^	2000	−4.7	3300	1.2
	Autosampler ^b^		−1.4		2.3
	Bench-top ^c^		1.5		−3.1
	Deep-freeze ^d^		5.7		8.0
	Long-term ^e^		4.0		0.0
Sorafenib N-oxide	Freeze–thaw ^a^	200	17.2	350	−5.8
	Autosampler ^b^		−9.2		−16.5
	Bench-top ^c^		26.5		23.1
	Deep-freeze ^d^		−5.1		−10.0
	Long-term ^e^		4.3		−0.8
Axitinib	Freeze–thaw ^a^	200	−3.7	350	−6.5
	Autosampler ^b^		−5.3		−3.3
	Bench-top ^c^		−7.9		−5.3
	Deep-freeze ^d^		−7.5		−7.2
	Long-term ^e^		3.0		4.9
Tivozanib	Freeze–thaw ^a^	200	9.3	350	3.9
	Autosampler ^b^		−6.3		−7.6
	Bench-top ^c^		−3.1		−2.9
	Deep-freeze ^d^		−2.6		−3.9
	Long-term ^e^		3.9		5.1
Sunitinib	Freeze–thaw ^a^	200	0.6	350	4.7
	Autosampler ^b^		0.1		5.1
	Bench-top ^c^		1.2		0.0
	Deep-freeze ^d^		−0.7		6.6
	Long-term ^e^		9.0		−0.3
Desethyl-sunitinib	Freeze–thaw ^a^	200	−0.6	350	0.0
	Autosampler ^b^		1.8		4.8
	Bench-top ^c^		5.0		3.8
	Deep-freeze ^d^		5.8		5.9
	Long-term ^e^		4.2		3.1

^a^ Freeze–thaw (three cycles, −80 °C vs. room temperature); ^b^ autosampler (10 °C, 24 h); ^c^ bench-top (room temperature, 24 h); ^d^ deep-freeze (−80 °C, five weeks); ^e^ long-term (−80 °C, five years); ^f^ relative deviation from the baseline concentration (RE%).

## Data Availability

The data presented in this study are available on request from the corresponding author due to privacy and legal reasons (specifically, restrictions under the Danish Data Protection Act and the European General Data Protection Regulation).

## Abbreviations

The following abbreviations are used in the manuscript:

ESI	Electrospray ionization
IS	Internal standards
LC-MS/MS	Liquid chromatography–tandem mass spectrometry
LLOQ	Lower limit of quantification
MRM	Multiple reaction monitoring
TDM	Therapeutic drug monitoring
TKI	Tyrosine kinase inhibitor
VEGFR	Vascular endothelial growth factor receptor
